# Impact of kidney volume on incidence of in-hospital kidney-related adverse outcomes in patients with acute heart failure

**DOI:** 10.1186/s12872-025-04502-4

**Published:** 2025-01-28

**Authors:** Akira Saito, Taku Asano, Nobuyuki Komiyama, Sachiko Ohde

**Affiliations:** 1 Department of Cardiology, St. Luke’s International University, Tokyo, Japan; 2 Graduate School of Public Health, St Luke’s International University, Tokyo, Japan; 3 Department of Cardiovascular Medicine, St. Luke’s International Hospital, Akashi-Cho 9-1, Chuo-Ku, Tokyo, Japan

**Keywords:** Heart failure, Cardio-renal syndrome, Organ size, Computed tomography

## Abstract

**Background:**

Recent studies revealed an association between small kidney volume and progression of kidney dysfunction in particular settings such as kidney transplantation and transcatheter aortic valve implantation. We hypothesized that kidney volume was associated with the incidence of kidney-related adverse outcomes such as worsening renal function (WRF) in patients with acute heart failure (AHF).

**Methods:**

This study was a single-center retrospective cohort study. It included patients admitted for AHF treatment between 2011 and 2021 and who underwent computed tomography (CT) that included images of the kidneys on the date of admission. We measured the volume of the right and left kidneys using dedicated volume analyzing software for 3D-CT (SYNAPSE VINCENT, Fuji Film, Tokyo, Japan) and determined the total kidney volume by adding the volumes of the left and right kidneys. We defined the composite of death from any cause, initiating renal replacement therapy, and WRF during hospitalization as major adverse kidney events (MAKE). We conducted multivariate logistic regression analysis to evaluate the impact of MAKE and each component of MAKE adjusted for age, sex, body surface area, estimated Glomerular Filtration Rate (eGFR) on admission date and the factors that were significantly associated with the incidence of MAKE by bivariate analysis.

**Results:**

In the 229 patients enrolled in the analysis, death from any cause, initiating RRT, and WRF occurred in 30 (13.1%), 10 (4.4%), and 85 (37.3%) patients, respectively. It was found that small kidney volume (≤ 250 ml) was independently associated with the increased incidence of MAKE (odds ratio 3.92, 95% confidence interval [1.18–13.08], *p* = 0.026) and WRF (odds ratio 6.58, 95%confidence interval [1.85–23.42] *p* = 0.004). The area under the receiver operating characteristic curve for multivariate logistic regression analysis of MAKE was 0.71.

**Conclusions:**

Kidney volume on admission was independently associated with the increased incidence of kidney-related adverse outcomes during hospitalization in patients with AHF.

## Introduction

Heart failure (HF) is defined as a condition in which the heart cannot pump the blood properly, resulting in symptoms such as difficulty breathing, leg edema, and fatigue. Due to systemic compensatory mechanisms, the symptoms can be mild. However, once these compensatory mechanisms fail, symptoms can worsen rapidly. This stage is called acute heart failure (AHF).Although HF treatment has made advances, about 2–17% of HF patients admitted to hospital die during hospitalization [1].

The incidence and prevalence of HF have been rapidly increasing worldwide. The global Burden of Disease Study reported that in 2017 about 64.3 million people were estimated to suffer from HF worldwide [2]. In the US, by 2030, the number of patients with HF is expected to increase by 44% compared to 2012 [3]. This global public health problem has been called a heart failure pandemic. Many researchers have addressed the challenges.

The interaction between the kidney and heart is one of a hot topic. Researchers have proposed several mechanisms but none of these have been fully elucidated. It has been proposed that low cardiac output from an impaired heart decreases renal perfusion, congestion increases renal vein pressure, and HF stimulates the sympathetic nervous system and hormonal system [4, 5]. About 25% of patients with HF suffer from worsening renal function (WRF) during heart failure treatment [6]. Several studies reported the association between WRF and poor prognosis in patients with HF [7]. Researchers have studied strategies designed to protect against injurious kidney-heart interactions.

Recent studies revealed an association between kidney volume measured by computed tomography (CT) and kidney function. Small kidney volume is a risk factor for low estimated glomerular filtration rate (eGFR) [8]. Patients with chronic kidney disease (CKD) often showed shrinkages of kidney due to nephron atrophy. Although kidney volume is related to age, gender, and body size [9–11], a small kidney is related to poor clinical outcomes in particular settings, after adjustment for these variables. In patients who had undergone transplantation, it has been reported that donor kidney size analyzed by CT reflected kidney function [12]. A cardiovascular study showed that kidney size predicted deterioration or improvement of kidney function after transaortic valve implantation [13]. In AHF settings, kidney size can differ from that observed in stable condition. AHF leads to kidney congestion, increasing the fluid volume within the tubular lumen, which accounts for a significant portion of the kidney's overall volume [14]. Additionally, AHF is associated with hemodynamic changes, including reduced renal artery blood flow. This lower blood supply may result in nephron damage and subsequent kidney shrinkage [15]. The impact of kidney size on renal function exacerbation in patients with AHF remains unclear. Several risk factors for worsening renal function (WRF), including age, diabetes, and baseline kidney function, are already well-known [15]. To the best of our knowledge, no study has examined the relationship between kidney size and WRF. We hypothesized that kidney volume might be a predictive indicator of kidney-related adverse events.

## Methods

### Subjects

We conducted a retrospective study by reviewing electronic medical records. The study recruited 229 consecutive patients admitted to the Cardiology Department at St Luke’s International Hospital, Tokyo Japan with AHF who underwent non-contrast CT that included images of their kidneys from January 1, 2011, to December 31, 2021. The diagnosis of AHF was based on the International Classification of Diseases (ICD) 10 code. We included patients with ICD codes I 50.0 – I 50.9 on admission. The exclusion criteria were as follows. 1) Patients who did not submit an informed consent form 2) Aged less than 18 years on the day of admission 3) Patients with an anatomical kidney abnormality 4) Dialysis patients. Anatomical kidney abnormalities comprised cystic kidney disease, kidney tumor, nephrectomized patients, solitary congenital kidney, acute renal infarction, or renal arteriovenous fistula. Dialysis patients included those undergoing continuous peritoneal dialysis or hemodialysis.

### Measuring kidney volume

**Fig. 1:**
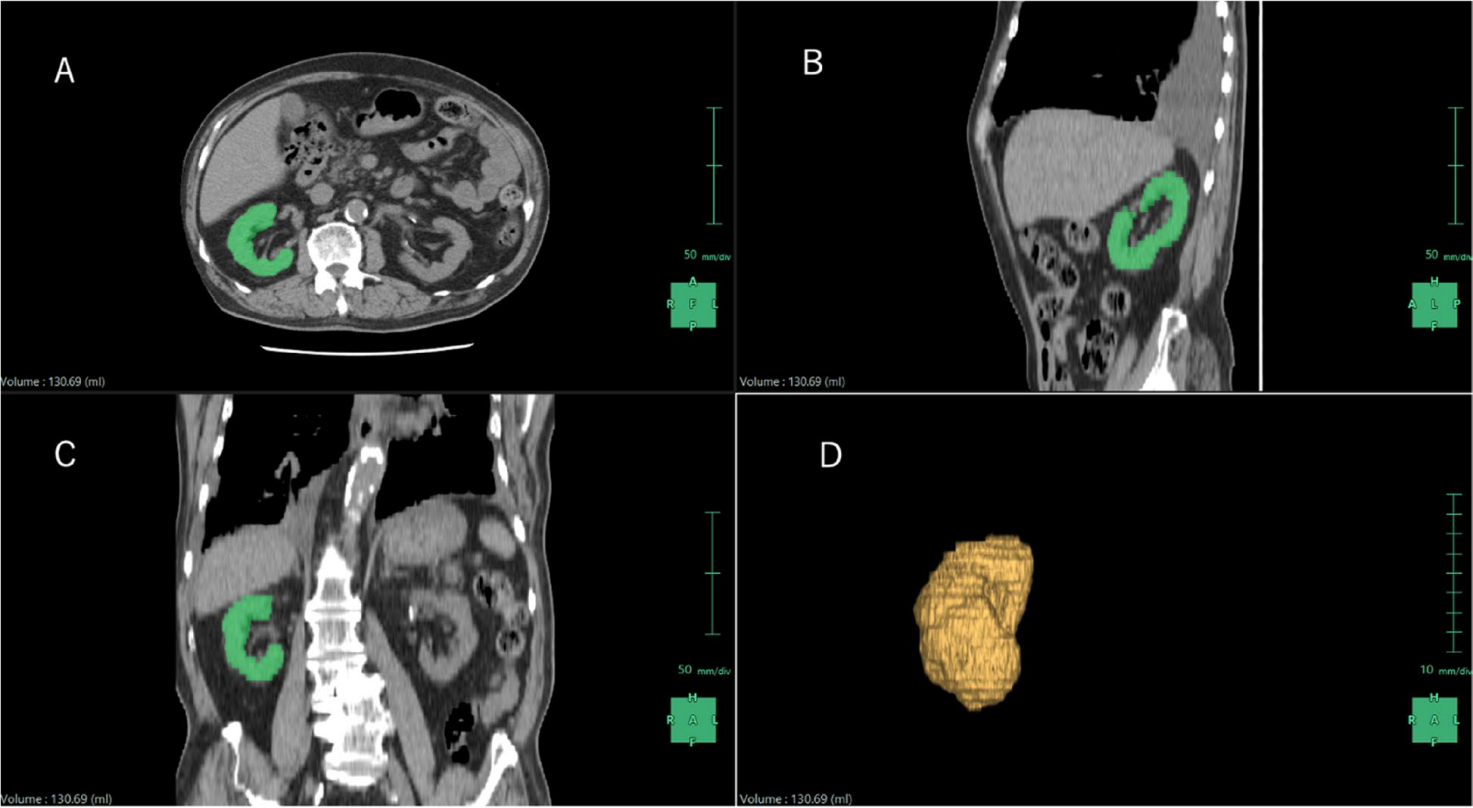
Measuring kidney volume by SYNAPSE VINCENT. Green color indicates a portion of the kidney. **A** Axial. **B** Sagittal. **C** Coronal. **D** 3D reconstruction

We analyzed non-contrast 5-mm slice CT data on the admission date and measured right and left kidney volume using 3D volume analyzing software called SYNAPSE VINCENT version 6.7. (Fuji Film, Tokyo, Japan). The kidney analysis function of the software automatically calculated the left and right kidney volumes separately (Fig. 1). The left and right kidney volumes were summed and defined as the total kidney volume.

### Data extraction and definitions

Patients’ demographic data such as age, height, body weight, systolic blood pressure, and past medical history obtained on the admission date were extracted from electronic medical records. Severe systolic blood pressure was defined as an initial blood pressure measurement of 160 mmHg or higher on admission. We calculated body surface area (BSA) using the Du Bois formula as follows. BSA (m2) = Height (cm)0.725 × Body weight (kg)0.425 × 0.007184. We collected the blood test data obtained on the admission date, or the first blood test performed after admission, except for creatinine. The physician in charge determined creatinine levels at the time he or she considered appropriate. We calculated estimated Glomerular Filtration Rate (eGFR) by using 2021 Chronic Kidney Disease Epidemiology Collaboration (CKD-EPI) creatinine equation [16]. WRF was defined as eGFR that had decreased by more than 20% from the admission date. We used the ejection fraction (EF) obtained during the first echocardiography following hospitalization. Heart failure with reduced EF (HFrEF) was defined as EF < 40%. We defined the composite of death from any cause, initiating renal replacement therapy, and WRF during hospitalization as major adverse kidney events (MAKE). We calculated the length of hospital stay for the MAKE group and non-MAKE group to ascertain know the impact of MAKE on the disease burden.

### Statistical analysis

We analyzed the kidney volume distribution and defined a small kidney as one with less than the median of total kidney volume. We checked the correlation between total kidney volume and the initial eGFR by using Pearson correlation analysis. The association between small kidney and the incidence of MAKE was evaluated by multivariate logistic analysis. We also analyzed the relationship between small kidney and each component of MAKE. We assessed the accuracy of the generated model by using the receiver operating characteristic (ROC) curve.

Continuous variables were expressed as mean ± standard deviation (SD) or medians and interquartile range (IQR). Categorical variables were expressed as percentages. Comparisons of continuous variables were analyzed using independent Student’s t-test or the Wilcoxon–Mann–Whitney test as appropriate, and comparisons of categorical variables were analyzed by Pearson’s χ2 test. The multivariate logistic analysis included total renal volume, the variables identified as risk factors by bivariate analysis, and the three variables already reported as being associated with renal volume: age, gender, and BSA. All hypotheses were analyzed by two-tailed tests, and a p-value of < 0.05 was considered statistically significant. STATA version 17.0 (STATA Corp, College Station, Tex) was used for all statistical analyses.

### Ethical statement

This study was conducted in accordance with the Declaration of Helsinki. We applied opt-out method to obtain consent on this study. Informed consent was obtained in the form of opt-out on the St Luke’s International Hospital website. The ethics committee at St Luke’s International Hospital approved the study protocol [Date: April 1 2022, 21‐R186].

## Results

### Baseline characteristics

**Fig. 2:**
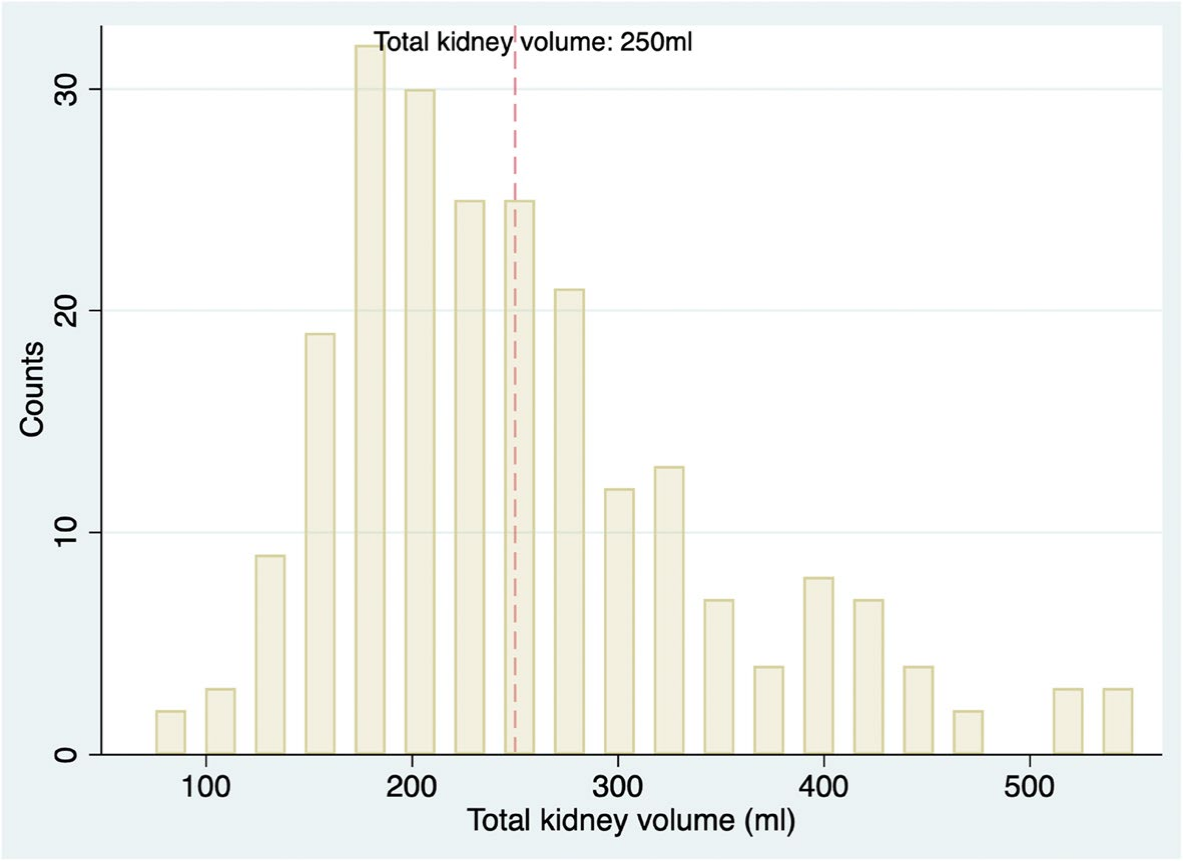
Distribution of total kidney volume at the date of admission

We analyzed 229 patients. As shown in Table 1, the mean age was 77.8 ± 14.3 years, and 118 (51.5%) of the patients were men. The median eGFR at the date of admission was 58.7 ml/min/1.73m^2^ (39.3–80.7 ml/min/1.73m^2^). The median N-terminal pro-brain natriuretic peptide (NTproBNP) was 5283 pg/ml (2596–12,345 pg/ml). There were 126 (55.0%) patients classified as HFrEF. Figure 2 shows the distribution of total kidney volume. The median total kidney volume was 236.2 ml (182.8–295.5 ml). We rounded this number to 250 ml and considered a volume less than 250 ml as indicative of a small kidney. The initial eGFR correlated with kidney volume (Pearson correlation analysis: coefficient = 0.40, *p* < 0.001). MAKE occurred in 102 (44.5%) patients. Death from any cause, initiating RRT, and WRF occurred in 30 (13.1%), 10 (4.4%), and 85 (37.3%) patients respectively. We divided patients into two groups according to the presence of MAKE. Compared to the non-MAKE group, the MAKE group had a higher percentage of small kidney (66.7% vs. 52.8%, *p* = 0033), lower eGFR on the date of admission (median 50.3 ml/min/1.73m^2^, IQR 31.3 to 70.8 ml/min/1.73m^2^ vs. 63.6 ml/min/1.73m^2^, IQR 41.8 to 87.4 ml/min/1.73m^2^ *p* = 0.001), higher troponin T (median 0.066 IU/L, IQR 0.035 to 0.15 IU/L vs. 0.033 IU/L, IQR 0.023 to 0.065 IU/L *p* < 0.001), higher NTproBNP (median 8112 IU/L, IQR 3754 to 17,585 IU/L vs. 3651 IU/L, IQR 1885 to 8136 IU/L *p* = 0.001) and longer duration of hospitalization (median 18 days, IQR 11 to 29 days vs. median 12 days, IQR 6 to 25 days, *p* = 0.006).

**Table 1:** Patient characteristics

	All (*N*=229)	non-MAKE (*N*=127)	MAKE (*N*=102)	*p*-value
Age - yr	77.8±14.3	76.7±14.2	79.2±14.3	0.19
Men - %	118 (51.5%)	62 (48.8%)	56 (54.9%)	0.36
Hight - cm	156.7±13.6	157.1±15.0	156.2±11.6	0.71
Body weight - kg	57.4±17.3	57.3±18.5	57.5±15.7	0.93
BSA - m^2^	1.58±0.26	1.58±0.27	1.58±0.25	0.98
Small kidney (total kidney volume ≤ 250ml) - %	135 (59.0%)	67 (52.8%)	68 (66.7%)	0.033
eGFR on admission date (IQR) - ml/min/1.73m^2^	58.7 (39.3-80.7)	63.6 (41.8-87.4)	50.3 (31.3-70.8)	0.001
Hypertension - %	167 (72.9%)	95 (74.8%)	72 (70.6%)	0.48
Diabetes - %	76 (33.2%)	42 (33.1%)	34 (33.3%)	0.97
Dyslipidemia - %	85 (37.1%)	49 (38.6%)	36 (35.3%)	0.61
Hyperuricemia - %	44 (19.2%)	28 (22.1%)	16 (15.7%)	0.23
Ischemic heart disease - %	55 (24.0%)	27 (21.3%)	28 (27.5%)	0.28
Congestive heart disease - %	102 (44.5%)	60 (47.2%)	42 (41.2%)	0.36
Perivascular disease - %	37 (16.2%)	22 (17.3%)	15 (14.7%)	0.59
Cerebrovascular disease - %	33 (14.4%)	16 (12.5%)	17 (16.8%)	0.35
Severe systolic hypertension (systolic blood pressure > 160mmHg) - %	72 (31.4%)	37 (29.1%)	35 (34.3%)	0.40
HFrEF (EF < 40%) - %	126 (55.0%)	71 (56.0%)	55 (53.9%)	0.76
Hemoglobin - g/dl	11.7±2.7	11.9±2.8	11.4±2.5	0.15
Hct - %	35.6±7.6	36.3±7.9	34.8±7.3	0.13
Serum sodium - mEq/L	138.0±5.2	138.0±4.9	138.0±5.6	0.95
Serum potassium - mEq/L	4.2±0.7	4.2±0.7	4.1±0.7	0.29
Serum cloride - mEq/L	103.7±5.8	103.7±4.8	103.7±6.8	0.97
Troponin T (IQR) - ng/ml	0.044 (0.026-0.11)	0.033 (0.023-0.065)	0.066 (0.035-0.15)	<0.001
N-terminal pro-brain natriuretic peptide (IQR) - pg/ml	5283 (2596-12345)	3651 (1885-8136)	8112 (3754-17585)	0.001
The length of hospital stay (IQR) - day	15 (8-27)	12 (6-25)	18 (11-29)	0.006

Plus minus values: mean ±SD. *IQR* interquartile range

### Association between small kidney and MAKE

**Fig. 3:**
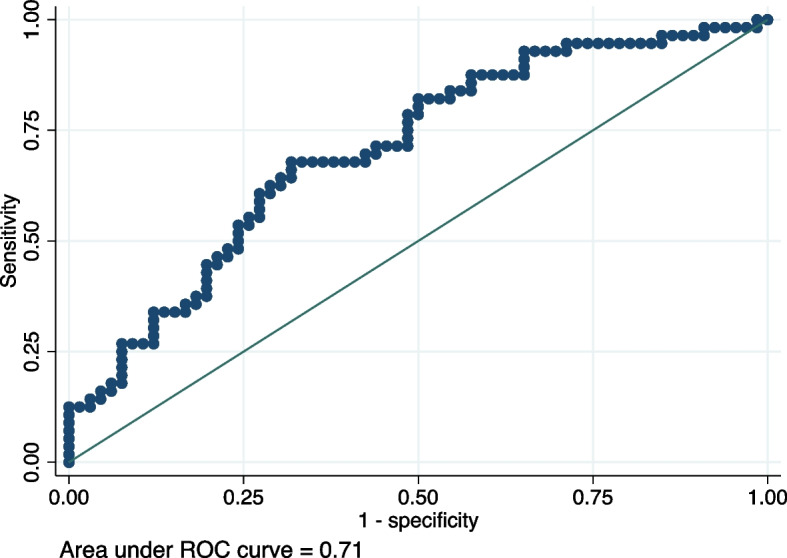
The ROC curve of the logistic analysis

Table 2 shows the result of multivariate logistic analysis for MAKE. In addition to age, gender, and BSA, which previous studies have already reported as being related to kidney volume, we also included the following variables that showed significant univariate differences as confounding factors: eGFR on admission date, troponin T, and NTproBNP. Small kidney was independently associated with the incidence of MAKE (OR = 3.92 [1.18–13.08], *p* = 0.026), adjusting for these confounding factors. We evaluated the association between the incidence of each component of MAKE and small kidney. Tables 3, 4 and 5. shows that small kidney was an independent risk factor for the incidence of WRF (OR = 6.58 [1.85–23.42], *p* = 0.004), not initiating RRT (OR = 6.66 [0.42–106.59], *p* = 0.18), nor death from any cause (OR = 0.26 [0.025 – 2.59], *p* = 0.25). The incidence of WRF was significantly associated with BSA (OR = 7.91 [0.78 – 80.09], *p* = 0.08). eGFR upon admission was significantly related to the incidence of RRT (OR = 0.91 [0.83 – 0.99], *p* = 0.02). The area under the ROC curve for multivariate logistic regression analysis of MAKE was 0.71 [0.61—0.80] (Fig. 3).

**Table 2:** Multivariate logistic analysis for the incidence of MAKE

The incidence of MAKE (*N*=102) (44.5%)	OR [95%CI]	*p*-value
Small kidney (total kidney volume<= 250ml)	3.92 [1.18-13.08]	0.026
Age - yr	1.00 [0.96-1.04]	0.94
Men	0.95 [0.37-2.46]	0.92
BSA - m^2^	5.17 [0.54-49.16]	0.15
eGFR on admission date (IQR) - ml/min/1.73m^2^	0.99 [0.98-1.01]	0.51
Troponin T - ng/ml	1.31 [0.44 - 3.86]	0.63
N-terminal pro-brain natriuretic peptide - pg/ml	1.00 [1.00 - 1.00]	0.31

**Table 3:** Multivariate logistic analysis for the incidence of WRF

The incidence of WRF (N=85) (37.3%)	OR [95%CI]	*p*-value
Small kidney (total kidney volume<= 250ml)	6.58 [1.85-23.42]	0.004
Age - yr	1.00 [0.96-1.04]	0.97
Men	0.89 [0.34-2.35]	0.81
BSA - m^2^	7.91 [0.78-80.09]	0.08
	0.99 [0.98-1.01]	0.46
Troponin T - ng/ml	0.81 [0.27 - 2.42]	0.71
N-terminal pro-brain natriuretic peptide - pg/ml	1.00 [1.00 - 1.00]	0.99

**Table 4:** Multivariate logistic analysis for the incidence of initiating RRT

The incidence of initiating RRT (N=10) (4.4%)	OR [95%CI]	*p*-value
Small kidney (total kidney volume<= 250ml)	6.66 [0.42-106.59]	0.18
Age - yr	1.00 [0.92-1.09]	0.93
Men	4.97 [0.30-81.52]	0.26
	1.60 [0.026-966.03]	0.89
	0.91 [0.83-0.99]	0.02
Troponin T - ng/ml	1.70 [0.13 - 22.05]	0.68
N-terminal pro-brain natriuretic peptide - pg/ml	1.00 [1.00 - 1.00]	0.19

**Table 5:** Multivariate logistic analysis for the incidence of death from any cause

The incidence of death from any cause (*N*=30) (13.1%)	OR [95%CI]	*p*-value
Small kidney (total kidney volume<= 250ml)	0.26 [0.025-2.59]	0.25
Age - yr	1.06 [0.96-1.16]	0.25
Men	0.51 [0.08-3.28]	0.48
	0.19 [0.0018-21.35]	0.49
	1.00 [0.97-1.03]	0.90
Troponin T - ng/ml	3.38 [0.88 - 12.98]	0.08
N-terminal pro-brain natriuretic peptide - pg/ml	1.00 [1.00 - 1.00]	0.22

## Discussion

We found that small kidney as measured by CT on the date of admission was significantly associated with the incidence of MAKE during hospitalization. For each component of MAKE, the incidence of WRF was strongly related to small kidney. Analysis using the ROC curve confirmed that our logistic model had good accuracy. Previous studies examining kidney size and clinical outcomes including WRF reported similar results. Small kidney from donors is associated with lower renal function as measured by scintigraphy following renal transplantation [12]^.^ The kidney function of patients with a small kidney tended to deteriorate after transaortic valve implantation [13].

A possible explanation for our result is that small kidney is related to impaired kidney function [17], which makes diuretic medications less effective [18, 19]. High-dose diuretics need to be used to relieve congestion and these medications are associated with the incidence of WRF in patients with heart failure [18, 19]. A study on patients with hepatocellular carcinoma reported a substantial correlation between kidney volume analyzed by 3D-CT and eGFR (r = 0.6) [17]. Our study revealed the smaller kidneys had lower eGFR, which suggested that deterioration of nephrons and other kidney structures reduced kidney volume. In a study examining the effect of certain diuretic medication used in heart failure, it was found that patients with smaller kidney size as measured by CT had a reduced response to the diuretic medication [20]. Deterioration of kidney structures may result in smaller kidneys, making diuretics less effective.

In this study, the eGFR at admission did not correlate with kidney-related adverse events even after multivariate adjustment, though several previous studies reported that eGFR at admission was associated with kidney-related adverse events [8]. The results could be attributable to the fact that acute heart failure often causes fluid overload, and the resulting volume expansion lowers the creatinine level by dilution. Swonlisky et al. determined that measuring serum creatinine is an inaccurate method for estimating kidney function in a patient with acute heart failure [21]. In contrast, kidney volume measured by CT at admission may be less affected by congestion and can predict kidney function and kidney-related adverse events more precisely in patients with acute heart failure compared to measuring the creatinine level at the time of admission.

We define small kidney as a total kidney volume of less than 250 ml using the median of sample kidney size. A previous study evaluating kidney size using MRI showed that the mean total kidney volume among normal subjects was 407 ml for men and 310 ml for women [22]. Previous studies that used CT obtained similar results [23]. The definition of a small kidney as 250 ml in our study was smaller compared with the results of previous studies. This may be because our study population was older and had poorer renal function compared to the normal population in previous studies.

The study revealed that the duration of hospital stay in the MAKE group was significantly longer compared to the non-MAKE group and that MAKE results in a higher disease burden. A previous study showed that although WRF was transient, it was associated with longer hospitalization and a higher risk of readmission and death [24]. The result suggests the importance of protecting against MAKE.

We used MAKE as a composite outcome for kidney-related adverse events. The concept was proposed by Frederic and Andrew, designed to parallel the term major adverse cardiac events (MACE) [25]. In 2024, Maeda reported that the number of articles evaluating MAKE has been increasing, reaching 204 publications by 2023 [26]. We applied MAKE because it can be easily measure and consistently applied across diverse patient populations.

To the best of our knowledge, this study is the first to reveal the association between kidney volume determined by CT upon admission and the incidence of MAKE. Our findings may predict the renal prognosis of heart failure patients at the time of admission. Early introduction of heart failure treatment that protects the kidneys such as sodium-coupled glucose transporter 2 inhibitor may result in a better prognosis for patients with small kidney volume on admission. Further works need to be done in this field.

The findings of this study need to be interpreted while taking several limitations into consideration. First, the method used to measure kidney volume using the CT application might not have been completely accurate. The accuracy of using SYNAPSE VINCENT to measure kidney volume has yet to gain wide acceptance. However, several studies reported the utility of SYNAPSE VINCENT for measuring organs. One study that estimated kidney volume from CT in a patient with autosomal dominant polycystic kidney disease found that using SYNAPSE VINCENT as the measurement tool strongly correlated with the conventional ellipsoid method [27]. In the setting of liver surgery, another study reported that there was a significant association between the actual weight of the resected specimen and the estimated volume obtained using SYNAPSE VINCENT [28]. It seems likely that the accuracy of SYNAPSE VINCENT will become more widely accepted. Second, the study was single-center retrospective observational study, and the sample size was small. RRT was initiated in only 10 patients in the study. We need to collect data from a larger sample. However, the study is valuable as the first report to evaluate CT-calculated kidney volumes and impact of volume on the kidneys during acute heart failure treatment. A multicenter large cohort study is needed to validate the results. Third, kidney size was influenced by several factors, including gender, age, BSA, and stenoses of renal arteries [11]. We considered these variables using multivariate logistic analysis, though we could not include every variable that has already been reported, such as stenoses of renal arteries.

## Conclusion

The study revealed that having a small kidney as measured by CT upon admission was the risk factor for kidney-related adverse events for patient with acute heart failure during hospitalization.

## Data Availability

The data that support the findings of this study are not openly available due to reasons of sensitivity and are available from the corresponding author upon reasonable request. Data are located in controlled access data storage at St Luke’s International Hospital.
